# AMPK Localization: A Key to Differential Energy Regulation

**DOI:** 10.3390/ijms222010921

**Published:** 2021-10-10

**Authors:** Qonita Afinanisa, Min Kyung Cho, Hyun-A Seong

**Affiliations:** Department of Biochemistry, School of Biological Sciences, Chungbuk National University, Cheongju 28644, Korea; qonita@chungbuk.ac.kr (Q.A.); tin1400@chungbuk.ac.kr (M.K.C.)

**Keywords:** AMPK, localization, CRM1, compartmentalization

## Abstract

As the central node between nutrition signaling input and the metabolic pathway, AMP-activated protein kinase (AMPK) is tightly regulated to maintain energy homeostasis. Subcellular compartmentalization of AMPK is one of the critical regulations that enables AMPK to access proper targets and generate appropriate responses to specific perturbations and different levels of stress. One of the characterized localization mechanisms is RanGTPase-driven CRM1 that recognizes the nuclear export sequence (NES) on the α subunit to translocate AMPK into the cytoplasm. Nuclear localization putatively employs RanGTPase-driven importin that might recognize the nuclear localization signal (NLS) present on the AMPKα2 kinase domain. Nucleo-cytoplasmic shuttling of AMPK is influenced by multiple factors, such as starvation, exercise, heat shock, oxidant, cell density, and circadian rhythm. Tissue-specific localization, which distributes AMPK trimers with different combinations, has also been shown to be vital in maintaining tissue-specific metabolism. Tissue-specific and subcellular distribution of AMPK might be attributed to differences in the expression of the subunit, the stabilization by protein regulators, tissue activity, and the localization of AMPK activators. Considering the importance of AMPK localization in coordinating signaling and metabolism, further research is due to fully elucidate the largely unknown complex mechanism underlying this regulation.

## 1. AMPK as a Key Regulator of Energy Homeostasis

Nutrient availability serves as a metabolic input to regulate gene and protein expression, as well as protein activity. AMP-activated protein kinase (AMPK) is a highly conserved serine/threonine kinase that links nutrient input to metabolic pathways to regulate energy homeostasis [1]. AMPK orthologs are present is almost all eukaryotes, serving a central role in regulating adaptive responses to starvation. In higher eukaryotes, such as mammals, AMPK is also utilized to regulate growth and metabolism in whole body as well as in specific tissues. AMPK monitors the change in AMP/ATP and ADP/ATP, and recovers energy levels by promoting catabolism to generate ATP and inhibiting anabolism to prevent further uses of energy [2]. AMPK is highly regulated by upstream kinases including liver kinase B1 (LKB1) in a nucleotide-dependent pathway, and calcium/calmodulin-dependent protein kinase kinase 2 (CAMKK2) in a nucleotide-independent pathway [3,4]. Recent studies also reported that TGF-beta-activated kinase 1 (TAK1) and A-T mutated protein (ATM) can act as AMPK kinases [5,6]. LKB1 is involved in AMPK regulation when the AMP level rises due to starvation, exercise, ischemia, and mitochondrial inhibitors, while CAMKK2 is involved in energy regulation related to redox status, and ubiquitination [7]. AMPK substrates include but are not limited to acetyl-CoA carboxylase (ACC), 3-hydroxy-3-methylglutaryl-Coa reductase (HMG-CoA), hormone-sensitive lipase (HSL), endothelial nitric oxide synthase (eNOS) and malonyl-CoA decarboxylase (MCD), CREB-regulated transcription coactivator 2 (CRTC2), histone deacetylases (HDACs), hepatocyte nuclear factor 4 alpha (HNF4a), carbohydrate response element-binding protein (ChREBP), sterol regulatory element-binding transcription factor 1 (SREBP1), 3-hydroxy-3-methylglutaryl-CoA reductase (HMGCR), glutamine-fructose-6-phosphate transaminase (GFAT1), GYS1, GYS2, Pparg coactivator 1 alpha (PGC1α), forkhead box O3 (FOXO3), mammalian target of rapamycin (mTOR), and regulatory-associated protein of mTOR (Raptor) [1,7,8,9,10,11,12,13,14,15,16,17,18,19,20]. AMPK activation will promote catabolic reaction including glycolysis, fatty acid oxidation, glucose uptake, autophagy, mitophagy, and mitochondrial fission to increase ATP levels [2]. AMPK can also inhibit anabolic reactions that utilize ATP, such as the synthesis of protein, fatty acid, sterol, glycogen, and rRNA [2].

## 2. AMPK Structure

**Figure 1:**
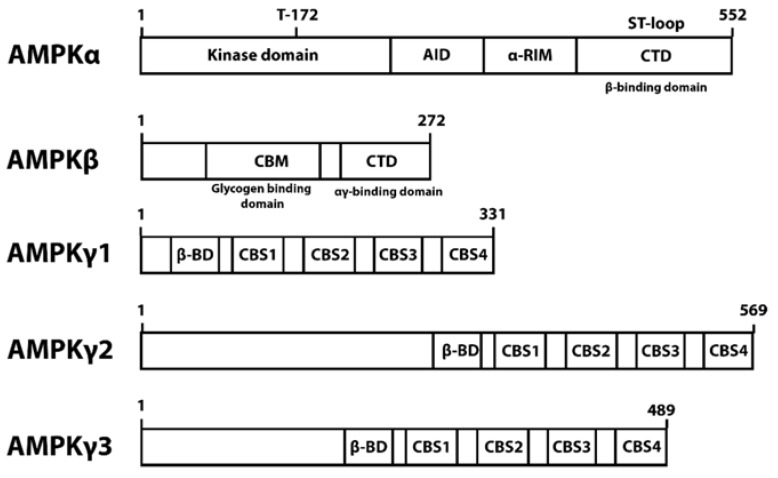
AMPK domains.

AMPK consists of catalytic unit α (α1 and α2), scaffolding unit β (β1, and β2), and regulatory unit γ (γ1, γ2, and γ3) (Figure 1). AMPK activation is achieved by phosphorylation of Thr172 in the kinase domain (KD) located at the N terminal of the α subunit. AMPKα also contains the regulatory autoinhibitory domain (AID), α regulatory-subunit-interacting motif (α-RIM), and serine/threonine-rich ST-loop on the C terminus domain (CTD). The scaffolding β subunit binds the α and γ subunit together using its C terminal domain [6]. The 85 residues of the β1 C terminal (186–270) bind the α1β1γ1 heterotrimer together with the support of the 313–473 residue of the α subunit. In addition, the 25 residues of the β1 C terminal (246–270) can bind the γ1, γ2, and γ3 subunit but is not sufficient to bind the α subunit [21]. The β subunit internal glycogen-binding domain senses glycogen, an inhibitor of AMPK [21]. The γ subunit is responsible for the allosteric activation of AMPK by AMP [21]. This allosteric activation was demonstrated to activate AMPKα2γ2 to a higher degree than AMPKα1γ1 [3,22,23], whereas AMPK with the γ3 subunit is the least activated [24].

AMPK subunit isoforms are expressed differentially across tissues and all combinations of trimers might form [2]. Studies on the roles of AMPK isoform combinations are still limited. However, it was suggested that they might have distinct biochemical properties, subcellular localization, stimulus, and responses [2,25].

To accommodate AMPK’s role as a central node between energy perturbations and various metabolic pathways, AMPK subunit isoforms are expressed differentially within subcellular compartments and among different tissues [22,26]. In addition, AMPK heterotrimers can translocate between organelles to modulate different responses upon different stimulus [7,18,27].

## 3. AMPK Localization

Eukaryotic cells are composed of subcellular compartments that together elaborate biochemical processes to support biological functions [28]. Subcellular localization of protein, particularly enzymes, is critical for its proper function in the cell. Localization allows proteins to access and interact with desired targets, and prevents unwanted reactions from occurring [29].

AMPK requires localization to regulate energy homeostasis in different subcellular compartments. Increased nuclear translocation can be expected to increase the activation of AMPK’s nuclear substrates and reduce the phosphorylation of cytoplasmic substrates, thus differentially regulating specific reactions to balance the metabolic perturbations. Under normal conditions, AMPK shuttles between the nucleus and cytoplasm to regulate its target in both compartments. During severe stress, AMPK nuclear localization might be modulated through inhibition of the nuclear export pathway [29], restricting AMPK to directly activate transcription at the DNA level. A combination of reduced nuclear export, increased nuclear import, and increased nuclear retention might also contribute to AMPK nuclear accumulation [7]. The redistribution might be attributed to balanced nuclear export and import, AMPKβ1 dephosphorylation, as well as β subunit myristoylation that anchors AMPK on the cytoplasm [3,7,13]. While AMPK trimers with β2 are transported to the nucleus, myristoylated AMPKα2β1 was found to phosphorylate acetyl-CoA carboxylase (ACC) in the cytoplasm, thus promoting fatty acid oxidation [30]. AMPK also activates ATGL in the cytoplasm to increase lipolysis, and inhibits mTOR to regulate autophagy and protein synthesis [31]. Other AMPK’s cytoplasmic target is the RNA-binding protein HuR, which increases the stability and transcription of its mRNA targets. The AMPK level was reported to be inversely associated with the levels of HuR and its binding to p21, cyclin B1, and cyclin A mRNA [12]. 

AMPKα2β2 rapidly translocates to the nucleus to increase peroxisome proliferator-activated receptor α (PPARα) expression upon stimulation by leptin [6]. AMPKα2β2 nuclear translocation seems to be influenced by a nuclear localization signal, and the Thr172 phosphorylation in the α2 subunit [6]. Environmental perturbations, such as heat, oxidant, and starvation, can induce nuclear accumulation of AMPK [29]. AMPK translocation to the nucleus is essential to regulate protein and gene expression either by interacting with transcription factors or histone deacetylases [26]. For example, AMPKα2 localization in the nucleus increases glucose transporter type 4 (GLUT4) production, which enhances glucose transport [26]. In the nucleus, AMPK regulation on various pathways might be connected with p300/cAMP-regulated enhancer-binding protein (CREB)-binding protein (p300/CBP), a transcriptional co-activator that integrates signaling and transcription apparatus to regulate gene expression [7,32]. AMPK regulates p300–CBP interactions with transcription factors by phosphorylating the protein on Ser89 [7,32]. Activated p300/CBP acetylates multiple downstream substrates, such as FOXO family, p53, and NF-kB [7,32]. FOXO, for example, will further cascade the signaling to regulate oxidative stress response, apoptosis, autophagy, cell cycle arrest, and metabolism [33].

Studies about AMPK homolog in yeast demonstrated that the β subunit homologs are localized in subcellular compartments and direct the translocation of the catalytic subunits. This suggests that AMPK localization is a conserved molecular phenomenon [34].

## 4. AMPK Nuclear Shuttling via RanGTPase-Dependent CRM1

The transport system in higher eukaryotes includes three functions: nuclear pore complexes (NPCs), adapter molecules or nuclear transport receptors (NTRs), and a coupling mechanism including the RanGTPase system [35]. Large molecules can cross NPCs by binding with NTRs. Cargo binding with NTRs accelerates shuttling through NPCs by a factor of 100 to >1000 [36]. 

Ran-GTPase-dependent receptors are the largest class of NTRs. The superfamily can be further categorized into import mediators (importin), export mediators (exportin), and carriers with both functions [35]. The RanGTPase system supplies the energy needed to drive the transport [35]. RanGTP binding will induce the substrate to bind to NTRs and transport them across nuclear pore complexes (NPCs) to the cytoplasm. In the cytoplasm, GTP will be hydrolyzed, and the RanGTP-exportin-substrate complex will disassemble. Regulator of chromosome condensation 1 (RCC1) exchanges GDP with GTP on Ran to maintain the supply of RanGTP [37]. Chromosomal maintenance 1 (CRM1), also called Exp1 and Xpo1p, is a versatile exportin that transports a wide range of cargoes from the nucleus to the cytoplasm [38]. CRM1 recognizes the leucine-rich nuclear export signal (NES) sequence on the proteins including AMPK [29]. Having an NES sequence on the α subunit, AMPK is one of the CRM1 substrates for shuttling across the nuclear membrane into the nucleus in a RanGTPase-dependent pathway [7,29,39]. Indeed, CRM1 inhibition using leptomycin B drug leads to nuclear accumulation of total AMPKα and β [7]. Besides recognizing the NES sequence, CRM1 can also recognize the three-dimensional structural conformation of protein [35]. However, whether this structural recognition function is also involved in AMPK shuttling would need further confirmation. This review also does not rule out the possibility of AMPK nucleo-cytoplasmic translocation via other currently unknown systems.

**Figure 2:**
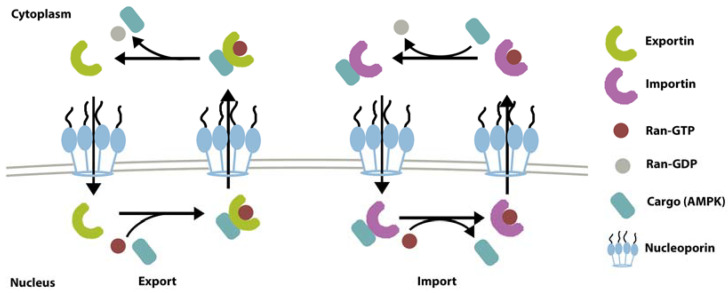
Mechanism of nuclear export and import with exportin and importin [44,45].

The Ran-GTPase system might also be involved in AMPK shuttling from the opposite direction, i.e., from the cytoplasm to the nucleus. This type of translocation is mediated by a family of proteins called importin, which recognizes a nuclear localization signal (NLS) sequence located on AMPKα2’s catalytic domain [6,40,41,42,43]. However, there has not been direct evidence for this, and the mechanism remains putative. An illustration of nuclear shuttling via exportin (CRM1) and importin is depicted in Figure 2. 

## 5. AMPK Domain Functions and Localization

### 5.1. AMPKα

**Figure 3:**
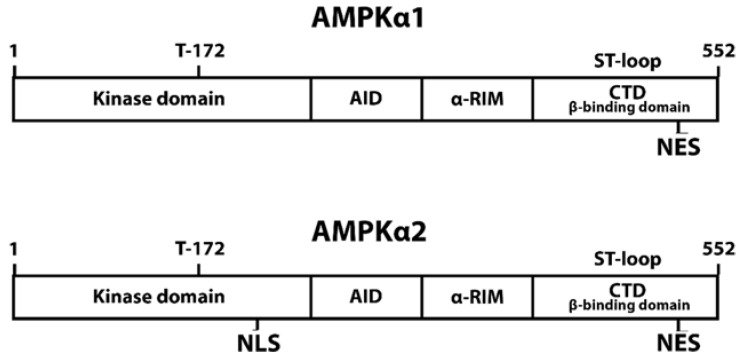
Localization-related sequences on AMPKα.

AMPKα is essential for cytoplasmic and nuclear localization as the subunit contains sequences recognized by NTR for subsequent transport. A previous study demonstrated that a highly conserved sequence in the C terminus of AMPKα is not necessary for binding the β and γ subunit. This part might act as a nuclear export sequence that is critical for subcellular localization and phosphorylation [29]. An NES located at the C terminal with the specific sequence of (ϕ-x_2/3_-ϕ-x_2/3_-ϕ-x-ϕ (ϕ: leucine, isoleucine, phenylalanine, valine, and methionine)), which closely resembles a CRM1-dependent NES, appears to be essential for AMPK cytoplasmic localization (Figure 3) [7,29,39]. The NES sequence seems to function via the Ran-dependent import pathway [29]. However, the leucine-rich sequence NES is conserved in both AMPKα1 and α2 [39], suggesting that differential localization between the two isoforms might be attributed to other mechanisms [29].

As for the nuclear localization, a study proposed that AMPKα2 translocation to the nucleus in response to leptin is dependent on the NLS sequence on the α2 subunit [6]. The AMPKα2 catalytic domain contains a conserved sequence of Lys-(Lys/Arg)-x-(Lys/Arg) located at Lys223 to Arg226 that fulfills the criteria of the monopartite classical NLS (cNLS) [46]. The mechanism of protein transport mediated by cNLS employs the importin family [47]. The NLS sequence is firstly recognized by the α subunit of importin. Cargo-bound importin α will subsequently be detected and bound by importin β subunit, forming a trimeric complex. The nuclear translocation of this complex is driven by the Ran-GTPase system through nucleoporin Nup153. Phosphorylation of Thr172 on AMPKα2 is reported to be important for its nuclear translocation during leptin stimulation [6]. The change in conformation upon phosphorylation might be required for the NLS sequence to be exposed and recognized by importin. Previous studies have not implicated importin in the regulation of AMPK nuclear translocation. However, temporarily interfering with Ran-GTP hydrolysis has also been shown to restrict AMPKα in the cytoplasm, suggesting nuclear localization of AMPK is also Ran dependent [29]. This and the presence of the monopartite cNLS sequence on AMPKα2 suggests that importin might also have a role in the nuclear translocation of AMPK.

AMPKα1 does not contain the NLS sequence, thus enabling differential localization between AMPKα1 and AMPKα2 [6]. However, AMPK localization can still be observed in organisms that do not produce leptin, indicating that other mechanisms might be involved [29]. It is also important to note that the effect of Thr172 phosphorylation to AMPK localization might not be universal during all stimuli. Kodiha et al.’s (2007) research on AMPK nuclear localization during various stresses shows that starvation resulted in cytoplasmic localization of phosphorylated AMPK, while heat shock and oxidant exposure localized dephosphorylated AMPK to the nucleus [7]. This is in contrast with Suzuki et al.’s (2007) observation that Thr172 phosphorylation induces nuclear export, while relocation to the cytoplasm depends on Thr172 dephosphorylation during leptin stimulation [6]. Leptin is a hormone that induces satiety and inhibits hunger. Adipose tissue produces leptin during excess nutrient intake as a signal that the energy need has been fulfilled and we can stop eating [6]. Leptin treatment simulates the condition of excess energy. The contrasting reports of AMPK activation’s effect on localization might indicate that AMPK localization is regulated differently during different energy states.

### 5.2. AMPKβ and γ

The AMPKβ subunit does not contain a localization signaling sequence recognized by NTR. However, AMPKβ post-translational modification might determine AMPKα’s availability to interact with exportin or importin. AMPKβ’s C terminus, which is essential to bind α and γ subunit, is highly conserved. Variations within the β isoform family are mostly present in the N terminus, which houses the sites of post-translational modification, such as myristoylation or phosphorylation. These modifications have been shown to be important in modulating cellular localization of AMPK [13]. 

Myristoylation of the β subunit anchors AMPK to cytoplasmic organelles, and stabilizes the heterotrimer conformation [13]. Previous study suggested that the initiation of AMPKα Thr172 phosphorylation requires that the β subunit is myristoylated. The important role of β subunit myristoylation shows that AMPK localization to the membrane is required for signal transduction [3]. Meanwhile, phosphorylation of α and β subunits in concert might be required to maximize AMPK activation [13]. The β subunit can be phosphorylated (S24 or S25 on both the β1 and β2 subunit, and S108 and S182 on the β2 subunit) by autophosphorylation or exogenous AMPK kinase acting on the β subunit [13]. Phosphorylation on S24, S25, and S182A of the β subunit is also linked with cellular localization of AMPK [13].

AMPKγ also does not contain the NTR-binding sequence. However, other proteins can recruit the AMPK trimer to a subcellular location by interacting with the γ subunit. Plectin can recruit AMPKα1γ1 to the Z discs of muscle cells by binding to the CBS domain of AMPKγ1 [48], suggesting the importance of the γ subunit in AMPK localization regulation. In addition, the N terminal domain is reported to be present in AMPKγ2 (569 aa) and AMPKγ3 (492aa) but not in AMPKγ1 (331 aa) (Figure 1) [23], allowing differential interaction with regulatory proteins. The γ1 subunit is also essential for Thr172 phosphorylation and nuclear translocation [13].

## 6. Factors Influencing the Regulation of AMPK Localization

### 6.1. Energy Perturbation

#### 6.1.1. Starvation

Serum starvation was reported to reduce and change the AMPK distribution in the nucleus and cytoplasm. Both total and phosphorylated AMPKα subunits still predominantly reside in the cytoplasm [7]. However, the amount of phosphorylated AMPKα increases in the nucleus while total AMPK decreased, suggesting that there are more signaling pathways in the nucleus that requires AMPK activation during starvation [7]. The functions regulated via AMPK during various stimulations are summarized in Table 1. In the cytoplasm, AMPK inhibitory phosphorylation suppresses the activity of acetyl-CoA carboxylases (ACC1 and ACC2) and HMG-CoA reductase (HMGCR), which are involved in lipid and cholesterol synthesis, respectively [1,19]. AMPK also suppresses the β-linked *N*-acetylglucosamine modifications of protein and hexosamine synthesis via glutamine-fructose-6-phosphate transaminase (GFAT1), and inhibits glycogen storage via glycogen synthases (GYS1 and GYS2) [8,20,41].

During overlong starvation, AMPK also inhibits gluconeogenesis [15]. In the nucleus, AMPK-direct phosphorylation inhibits the transcriptional activity of class IIA histone deacetylases (HDACs) and (CRTC2), which are the upstream regulator of the gluconeogenesis pathways FOXO and CREB, respectively. Lipid and glucose metabolisms are also controlled at the transcriptional level by AMPK via hepatocyte nuclear factor 4α (HNF4α), carbohydrate-responsive element-binding protein (ChREBP), and sterol regulatory element-binding protein1 (SREBP1) [15]. AMPK phosphorylation activates HNF4α, which subsequently will activate PPARα, a positive regulator of beta oxidation [40].

In the nucleus, AMPK phosphorylates ChREBP at Ser568, thereby reducing its DNA binding affinity [42]. The ChREBP signaling pathway is the link integrating glucose and lipid metabolism [17]. ChREBP upregulates liver pyruvate kinase (L-PK) at the terminal step of glycolysis, thus increasing the pyruvate level to feed the tricarboxylic acid cycle. Furthermore, it also stimulates the expression of essential lipogenesis-related enzymes, such as ATP citrate lyase (ACL), ACC, fatty acid synthase (FAS), and stearoyl-CoA desaturase-1 (SCD1) [17]. Thus, AMPK inhibitory phosphorylation on ChREBP leads to suppression of lipid synthesis.

AMPK also induces autophagy by activating FOXO3, unc-51-like kinase 1 (ULK1), and Beclin1, as well as by inhibiting mTORC1 via tuberous sclerosis 2 (TSC2) during starvation [15]. FOXO3 is a transcriptional regulator of autophagy-related genes. AMPK and FOXO3 interaction occur in two steps. In the cytoplasm, AMPK reduces mTOR/AKT signaling, thus promoting FOXO3 dephosphorylation. Dephosphorylated FOXO3 will translocate to the nucleus, where AMPK directly phosphorylates FOXO3 to induce the transcription of gamma-aminobutyric acid receptor-associated protein-like 1 (GABARAPL1), Beclin1, and microtubule-associated protein 1 light chain 3 beta (LC3B) [11]. GABARAPL1 and Beclin1 are proteins involved in autophagy initiation while LC3B is essential in autophagosome expansion and specific degradation [11,15,43].

During stress, phosphorylation of AMPKα1/2 was observed to be inversely correlated with extracellular signal-regulated kinase 1/2 (ERK1/2) activation and the two pathways may interact via both positive and negative feedback. During nonstress condition, the interfering mitogen-activated protein kinase (MEK)-ERK1/2 pathway also results in nuclear localization of AMPKα and β [7,29].

In addition, the degree of cellular AMP elevation, which might fluctuate depending on the severity of nutrient and energy stress, can activate distinct pools of compartmentalized AMPK [18]. This regulation enables AMPK to activate different sets of targets as appropriate response to different levels of stress. A low increase of AMP stimulates AMPK activation in lysosomes through the AMP-dependent axis inhibition protein (AXIN)-based pathway to reduce anabolism and increase catabolism via ACC1, SREBPc, and other proteins. A moderate AMP increase results in the activation of AMPK in the cytoplasm also via the AMP-dependent AXIN pathway. A high increase of AMP increases AMPK activation in all compartments in an AXIN-independent manner. AXIN is a scaffold protein that brings LKB1 and AMPK together in a complex at the lysosome [18].

#### 6.1.2. Exercise

AMPK is activated during exercise in human skeletal muscle, and liver and adipose tissue of rodents [49]. This will signal the cells to increase glucose transport, glycolysis, and fatty acid oxidation to promote ATP production, and reduces ATP-consuming processes, such as lipid and protein synthesis. In skeletal muscle, CAMKKK2 is surmised to act as the upstream activator of AMPK, especially for AMPKα1 [50].

Exercise with prolonged low intensity or an intensity of 60% over the maximum aerobic capacity is reported to induce AMPK activation [49]. Among the three AMPK trimers expressed in human muscle, AMPKα1β2γ1, α2β2γ1, and α2β2γ3, exercise only activates AMPKα2β2γ3 [51]. This complex activation is associated with increasing phosphorylation of ACC2. Thus, exercise inhibits lipid synthesis and promotes fatty acid oxidation [52]. During muscle contraction, Ca2+ signaling and active ERK regulate fatty acid oxidation [53]. ERK 1/2 is also suggested to regulate AMPK’s cytoplasmic translocation [7]. In addition, exercise inhibits ACC2 and promotes fatty acid oxidation that occur in the cytoplasm [15]. Together, it can be surmised that during exercise, AMPK is activated and localized at the cytoplasm. This is akin to the starvation effect to AMPK, both being a form of ATP depletion.

### 6.2. Heat Shock

Heat shock was shown to reduce phosphorylation and increase nuclear localization of AMPKα1/2 [7]. During stress, both AMPKα and β accumulate in the nucleus to upregulate the expression of proteins necessary for catabolism and energy production. Moderate heat stress of around 40 °C increases the expression of coactivator-a alpha (PGC-1α) on muscle cells in vitro [9]. PGC-1α is a direct substrate and transcriptional target of AMPK. AMPK phosphorylates PGC1α to increase its activation and regulate mitochondrial biosynthesis to increase the transcription of PGC1α via sirtuin-1 (SIRT1), a NAD-dependent deacetylase located in the nucleus [9,10].

Previous study demonstrated that AMPKα1/2 and β1/2 accumulate in the nucleus 2 to 3 h after heat stress and decrease after 5 h [7]. However, it was observed that the change in the nuclear/cytoplasmic ratio of total AMPKα1/2 is not always proportional to the ratio of the nuclear/cytoplasmic ratio of p-AMPKα1/2, indicating no direct link between phosphorylation and total AMPK localization. This might be due to the heat shock effects on the nucleocytoplasmic shuttling system. CRM1 inhibition has been shown to result in AMPK α and β nuclear accumulation [7]. There is not yet a report on high temperature effects on CRM1 and the RanGTPase system. However, it is plausible that heat shock might have compromised the function of either or both components of the Ran GTPase system, or other unknown transport systems, thereby localizing AMPK on the nucleus. This suggests that the distribution of total AMPKα1/2 and β 1/2 might involve more complex regulation [7].

### 6.3. Oxidant

Diethyl maleate (DEM)-induced oxidative stress results in nuclear translocation of total AMPKα [7]. However, AMPK phosphorylation was reduced in both the cytoplasm and nucleus, with a higher degree of phosphorylation observed in the cytoplasm [7]. One of the AMPK mechanisms to increase the cellular antioxidant potential is by increasing the expression of thioredoxin (Trx) via FOXO3. Phosphorylated FOXO3 recruits histone acetylase p300 to the Trx promoter in the nucleus and increases its expression [54]. Trx catalyzes the cysteine thiol-disulfide exchange to reduce oxidized proteins, thereby maintaining the redox balance in the cell. PGC1α activation by AMPK also promotes mitochondria regeneration to reduce ROS production [10].

### 6.4. Circadian Rhythm

The expression of AMPKβ2 was observed to follow a circadian rhythm with high production during the day. As localization is regulated by AMPK complexes’ composition, the rhythmic generation of AMPKβ2 would induce diurnal regulation of nuclear translocation. This was confirmed as the peak of AMPKα1 nuclear import synchronized with that of AMPKβ2. As a result, activation of downstream targets is also diurnally regulated. The phosphorylation of Raptor (Ser792) and ACC (Ser79) was reported to be higher during the day. In addition, AMPK also modulates the non-light-dependent biological clock by phosphorylating and thus destabilizing the clock component cryptochrome 1 (CRY1), indicating the complex and interconnected regulation between metabolism and the circadian rhythm [55].

### 6.5. Cell Density

**Figure 4:**
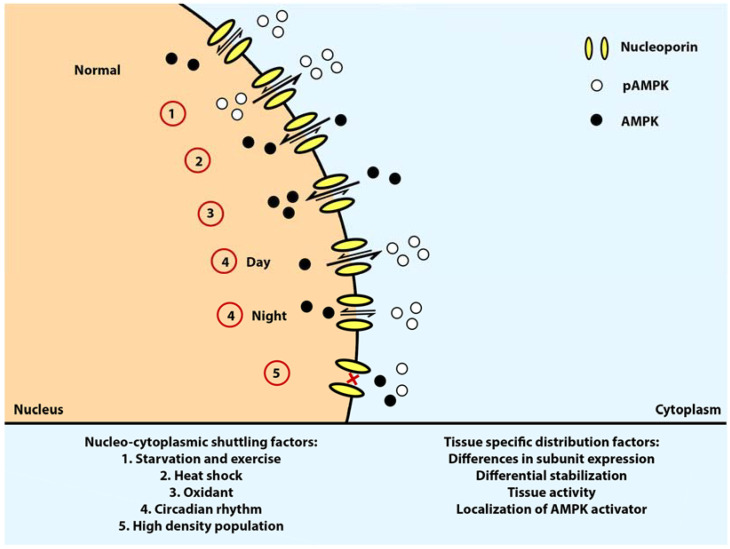
AMPK nucleo-cytoplasmic shuttling upon different stimulation. pAMPKs are more localized on the cytoplasm upon starvation, exercise, and during the day. Upon heat shock and oxidant exposure, AMPK undergoes nuclear localization in a dephosphorylated state. Arrow: distribution direction of total AMPK, white circle: Thr 172 phosphorylated AMPK, black circle: non phosphorylated AMPK, yellow channel: nucleoporin.

Distribution and shuttling of AMPK is regulated by cell density [7]. Both α and β subunits seem to be restricted in the cytoplasm in cell cultures with high density. This was proposed to be due to reduced nuclear import and increased AMPK subunits anchoring in the cytoplasm. The total amount and activation of AMPKα1/2 and ERK1/2 are also reduced in high-density cultures whereas the amount of AMPK β1/2 is increased, although the mechanism underlying this phenomenon has not been elucidated [7]. AMPK nucleo-cytoplasmic localization upon various stresses is illustrated in Figure 4. 

## 7. Tissue- and Subcellular-Specific Localization of AMPK

AMPK subunits exhibit heterogenous subcellular distribution or activation in different cell types or subcellular structures [13]. AMPK activation in specific structures allows the cell to phosphorylate protein targets precisely to incite an accurate respond to corresponding stimulation. For example, AMPKα was reported to transiently bind to mitotic structures during mitotic stages in epithelial cells, including centrosomes’ spindle poles, central spindle midzone, and midbody, to mediate protein phosphorylation needed for the process [56]. This differential distribution might be attributed to differences in the expression of the subunit, the stabilization by protein regulators, tissue activity, and localization of AMPK activators.

Tissue-specific localization can be a result of differential expression of the AMPK subunit isoforms. In neurons, β1 and β2 are predominant in the nucleus and cytoplasm, respectively. Meanwhile, in astrocytes, both β isoforms are both prevalent in the cytoplasm [13]. AMPKα2 and AMPKβ2 are present in low levels in many tissues but are predominant in skeletal and cardiac muscle [25]. Conversely, AMPKβ1 is expressed in a high concentration in the liver and low concentration in skeletal muscle [13]. The tissue-specific expression pattern of AMPK subunit isoforms might contribute to the differential physiological function. For example, AMPKα1 and AMPKα2 are more abundant in oxidative and glycolytic muscle fibers, respectively. In concordance, AMPKα1 is observed to be involved with aerobic respiration while AMPKα is more correlated with glycolysis and glucose metabolism [48,57,58].

Energy perturbation, such as exercise, can also stimulate tissue-specific AMPK localization as discussed in the previous section. It was reported that exercise increases AMPKα2 abundance in the nucleus in human skeletal muscle, leading to GLUT-4 expression and enhanced glucose transport [26].

Different isoforms of the subunit can also localize differently in the same tissue. In cardiomyocytes, the γ2 subunits alone might be transcribed to three different transcripts using different promoters. One transcript consists of the nucleotide binding domain (γ2-short), whereas the other two comprises the nucleotide binding domain and an N terminus of different lengths (γ2-long and γ2-3B) [23,59]. In cardiac muscle, the γ2 subunit, particularly the γ2-3B variant, was found to be the predominant γ subunit. The AMPK γ2-3B localized on cardiomyocytes in a striated pattern along the Z-disk. AMPKγ3 localized on the I band in the skeletal muscle, also aligning to the Z-disk. This suggests that AMPK trimers containing γ2-3B and γ3 might function similarly in different muscle tissues [59].

The composition of AMPK complex might be influenced by differential stabilization by regulatory proteins. Plectin, a cytolinker protein that is predominantly expressed in mitochondria-rich slow oxidative type 1 myotubes, binds to AMPKγ1 to stabilize AMPKα1γ1 complex, thus increasing this isoform combination presence in ratio to other complexes [48].

Subcellular localization of AMPK activators can also lead to localized AMPK activation in a particular structure. Boehlke et al. (2010) proposed that AMPK might undergo localized activation in the basal body of cilia to downregulate mTOR and control cell size [27]. Basal body is a modified centriole comprising ninefold microtubule-triplets that mediates the growth of cilium [60]. Cilium is a motile appendage produced by cells to drive fluid flows, sense the environment, and take part in signaling [60]. Specialized human cells produce multiple basal bodies and cilia, such as mucociliary epithelium or escalator. This type of cell is found along the airway from the nose, bronchi, and bronchioles to move disrupting objects into the throat [60]. Cilium is involved in regulating mTOR signaling by facilitating signaling molecules’ translocation through an intraflagellar transport system. Flow-induced cilia bending downregulates mTOR activity in controlling cell size. This mechanism might be regulated by LKB1 and AMPK as flow promotes AMPK phosphorylation by LKB1 in the basal body of cilia [27]. This interaction was reported to partly regulate insulin signaling to glucose control [61]. This localization regulation via LKB1 regulation has also been identified on lysosome via a scaffold protein called AXIN. AXIN binds to lysosome and LKB1, allowing the following localized activation of AMPK on lysosome [18].

Tissue-specific interference of AMPK has been shown to result in various ailments [27,59,62]. Overactivated AMPKα1 was observed to occur in the striatal neurons of humans and mice with Huntington’s disease, causing brain atrophy, loss of neuron cells, and rising levels of Htt aggregates [62]. The overactivation is a result of polyQ-expanded mutated Htt protein that induces the activity of CAMKK2, ultimately leading to tissue-specific overactivation of AMPKα1. This suggests that aberrant activation of AMPK can occur differentially as the disease’s pathogenesis, indicating the importance of tight regulation on AMPK localization to maintain proper cellular functions.

Further studies are due to illuminate all aspects of AMPK localization. Studies exploring the shuttling mechanism of AMPK in detail using a greater variety of stimulation can be helpful to define AMPK’s function in cell signaling. For example, the effect of phosphorylation on AMPK localization might be different during stresses or excess nutrient. In addition, direct evidence to confirm importin’s function in AMPK shuttling is still required. This information can be beneficial to exploit AMPK more accurately as a target of therapeutic drugs for diseases related to homeostasis and energy imbalance, such as diabetes, obesity, and hepatic steatosis. As the central node coordinating energy input and metabolism, understanding molecular dynamics and regulation underlying AMPK localization would overall benefit the studies of all proteins related to the metabolic pathway.

## Figures and Tables

**Table 1:** The distribution of total and phosphorylated AMPK and the protein target activated during different stimuli.

Stimuli	Phosphorylated AMPK Localization	Total AMPK Localization Distribution	Protein Target	Effect	References
Starvation	High in cytoplasm	High in cytoplasm	Nucleus:		[7]
			CRTC2	↑ gluconeogenesis	[14]
				↓ lipogenesis	
			HDACs	↑ gluconeogenesis	[15]
			HNF4a	↑ lipid and glucose metabolism	[17,18,40]
			ChREBP		
			SREBP1		
			Cytoplasm:		
			ACC1 and ACC2	↓ lipid synthesis	[19]
			HMGCR	↓ cholesterol synthesis	[1]
			GFAT1	↓ hexosamine pathway and O-GlycNacylation	[15,20]
			GYS1, GYS2 mTOR	↓ glycogen storage	[8,15]
Heat	Low in both cytoplasm and nucleus	High in nucleus	Nucleus: SIRT1 Cytoplasm:	↑ PGC1α expression Mitochondrial adaptation ↑ coupled and uncoupled respiratory capacity	[10] [7,9,10]
			PGC1α		
Oxidant	Low in both cytoplasm and nucleus	High in nucleus	Nucleus:	↑ Trx expression	[33]
			FOXO3		
			Cytoplasm:	↑ mitochondria biosynthesis ↓ ROS	[7,10,11]
			PGC1α		
Circadian rhythm (during the day)	High in cytoplasm	High in cytoplasm	Cytoplasm:	↓ lipid synthesis	[33]
			Raptor, ACC		

* up arrow (↑) indicates an increased function, down arrow (↓) indicates a decreased function.
